# Solutions to problems of nonexistence of parameter estimates and sparse data bias in Poisson regression

**DOI:** 10.1177/09622802211065405

**Published:** 2021-12-21

**Authors:** Ashwini Joshi, Angelika Geroldinger, Lena Jiricka, Pralay Senchaudhuri, Christopher Corcoran, Georg Heinze

**Affiliations:** 1 Department of Mathematics and Statistics, University of Helsinki, Helsinki, Finland; 2 Center for Medical Statistics, Informatics and Intelligent Systems, Section for Clinical Biometrics, 27271 Medical University of Vienna, Vienna, Austria; 3 Cytel Inc., Cambridge, MA, USA; 4 Jon M. Huntsman School of Business, Department for Data Analytics and Information Systems, 4606 Utah State University, Logan, UT, USA

**Keywords:** Count data, Firth’s penalization, generalized linear models, penalized regression, Poisson regression, separation

## Abstract

Poisson regression can be challenging with sparse data, in particular with certain data constellations where maximum likelihood estimates of regression coefficients do not exist. This paper provides a comprehensive evaluation of methods that give finite regression coefficients when maximum likelihood estimates do not exist, including Firth’s general approach to bias reduction, exact conditional Poisson regression, and a Bayesian estimator using weakly informative priors that can be obtained via data augmentation. Furthermore, we include in our evaluation a new proposal for a modification of Firth’s approach, improving its performance for predictions without compromising its attractive bias-correcting properties for regression coefficients. We illustrate the issue of the nonexistence of maximum likelihood estimates with a dataset arising from the recent outbreak of COVID-19 and an example from implant dentistry. All methods are evaluated in a comprehensive simulation study under a variety of realistic scenarios, evaluating their performance for prediction and estimation. To conclude, while exact conditional Poisson regression may be confined to small data sets only, both the modification of Firth’s approach and the Bayesian estimator are universally applicable solutions with attractive properties for prediction and estimation. While the Bayesian method needs specification of prior variances for the regression coefficients, the modified Firth approach does not require any user input.

## 1 Introduction

Poisson regression is widely used to model the distribution of count variables as functions of predictive covariates. This approach provides particular utility when accommodating differential follow-up times of study subjects,^1^ as well as the modelling of ‘non-events’ or excess zeroes through so-called zero-inflated models.^2^ As with other generalized linear models, such as logistic regression, Poisson regression can be especially challenging in the presence of rare events, making it more likely that particular covariate patterns in a given dataset result in the nonexistence of maximum likelihood (ML) estimates; for example, if no events are observed for one of two groups represented by a binary covariate. A necessary and sufficient condition for the nonexistence of ML estimates has been identified by Correia et al.^3^ This problem of ‘separation’ has been studied extensively for logistic regression,^4–7^ but comparatively little is known about how various approaches perform when separation arises in Poisson modelling, such as the bias reduction method of Firth^8,9^ and (in special cases) conditional exact Poisson (EP) regression.^10^ Bayesian alternatives, including the application of weakly informative priors for the distribution of the log relative risk, have likewise not been evaluated with regard to separation. Given the common use of Poisson regression, particularly for rare events and in other settings that are prone to separation, more definitive empirical studies are needed to assess the comparative performance of these modelling options. In particular, we need to better understand how various modelling choices impact the estimation of regression coefficients to ensure that analysts are confident in their predictions.

In this paper, we provide a more comprehensive evaluation of methods that provide finite estimates of regression coefficients when separation arises. We propose a modification of Firth’s approach to improving its performance for predictions, without compromising its attractive bias-correcting properties for regression coefficients. Furthermore, we include the conditional median unbiased estimator^11^ as implemented in the software package LOGXACT^10^ or in SAS’s PROC GENMOD and a Bayesian estimator using weakly informative priors in our evaluation. To provide context, we first illustrate the issue of separation for rare event data with a dataset arising from the recent outbreak of COVID-19. We subsequently provide a brief review of Poisson regression and describe proposed solutions to the problem of separation. We then summarize the results of a comprehensive simulation study to compare these options under a variety of realistic scenarios. We further illustrate their relative performance with an additional example.

## 2 Motivating example

During the outbreak of the coronavirus (COVID-19) in winter and spring 2020, employees of supermarkets, nursing homes, and hospitals were considered key professionals as they are in contact with many clients each day even under the lockdowns of public life that were imposed by many countries in that time. Many of their clients such as older adults or persons with chronic diseases were considered to be at high risk of a severe course of the disease if they got infected. At some point, the question arose if more stringent control of virus spread should be imposed on one of these groups, in particular, considering the unknown probability of asymptomatic infections. Therefore, on 28 March 2020, and 30 March 2020, a representative series of 1161 tests for the presence of an infection with the novel coronavirus (COVID-19) was performed in Austria among randomly selected asymptomatic employees of supermarkets, nursing homes, and hospitals.^12^

The research question behind this series was how the risk of infection differs between employees of supermarkets, nursing homes, and hospitals. The question could be answered by Poisson regression to compute risk ratios, for example, comparing nursing homes and hospitals to supermarkets.

Among the 1161 persons tested, only six tested positive for COVID-19. Three of them were working in hospitals and three in nursing homes (Table 1).

**Table 1.** Austrian COVID-19 test data.

Type of employment	Positive	Tested
Supermarket	0	352
Nursing home	3	444
Hospital	3	365

Unfortunately, neither SAS/PROC GENMOD nor R/glm was able to provide risk ratios based on a Poisson regression analysis because ML analysis failed as there was a category with no events. However, the conditional median unbiased estimates for the two interesting risk ratios and associated exact^13^ 95% confidence intervals (CIs) were 3.05 (0.46, ∞) for nursing homes versus supermarkets and 3.71 (0.56, ∞) for hospitals versus supermarkets. Using Firth’s bias reduction approach,^8^ which in the absence of covariates can be obtained by adding 0.5 events to each of the three observations, the analysis resulted in risk ratios (95% profile penalized likelihood (PPL) CI) of 5.55 (0.54, 746.13) and 6.75 (0.65, 907.60), respectively. Therefore, while it appears that employees of nursing homes or hospitals are at considerably higher risk of spreading the disease compared to supermarket employees, this claim is not fully supported by the study but still could be found in local media. Note that estimates of risk accompanied by 95% CI in each group, which could be obtained much easier than risk ratios, give a good summary of the data but do not answer the question of whether supermarket employees or health care workers are at higher risk of infection.

## 3 Methods

In this section, we will review the Poisson regression model with a special focus on nonexistence of ML estimates. We will further review Firth’s correction in the context of the Poisson regression model and we will propose a modification that gives unbiased predictions. Finally, we will present exact conditional analysis and Bayesian estimation with weakly informative priors.

### 3.1 The Poisson model

The Poisson model assumes that the counts of events in a study, *Y*, follow a Poisson distribution with parameter μ : Y∼Poisson(μ) , where the logarithm of μ is modelled by a linear combination of covariates: log(μ)=Xβ+Z . Here, *X* describes a n×(k+1) matrix of covariates, with *n* and *k* denoting the number of observations and covariates, respectively. By convention, $X_{\cdot 0}$ , the first column of *X*, consists of 1s only to enable the estimation of an intercept. *Z* denotes an offset variable, for example, the logarithm of follow-up time or the number of people tested in total. The parameter μ is interpreted as incidence per unit of follow-up time, $\beta_{0}$ is the intercept and $\beta_{j}$ , j=1,…,k , is the log incidence rate ratio (IRR) between two individuals differing in $X_{j}$ by one unit.

The log-likelihood of the Poisson model is given by $\ell (\beta )=\overset{n}{\underset{i=1}{\sum }}\;[-\exp\;(x_{i\cdot }\beta +z_{i})+(x_{i\cdot }\beta +z_{i})y_{i}-\log\;(y_{i}!)].\;$ Estimates of $\beta_{j},$ j=0,…,k , can be obtained by ML estimation, solving the score equations $U_{j}(\beta )=\sum_{i=1}^{n}x_{ij}[y_{i}-\exp\;(x_{i.}\beta +z_{i})]=0$ for j=0,…,k , where $x_{ij}$ is the observed value of covariate *j* for subject *i*, $x_{i\cdot }$ is the row vector of covariate values for subject *i*, and $z_{i}$ is the value of the offset for that subject.

### 3.2 Conditions for nonexistence of ML estimates in Poisson regression and consequences

In the coronavirus testing study, no infections were detected among the 352 supermarket workers, while among the 365 hospital employees, three were infected. The risk ratio would be computed as (3 of 365)/(0 of 352), which is not defined because of the division by zero. Similarly, there is no finite maximizer β of the corresponding Poisson likelihood. Correia et al.^3^ showed that in Poisson regression the ML estimate does not exist if and only if there is a non-zero (k+1) -dimensional vector $\gamma^{*}$ such that $x_{i\cdot }\gamma^{*}=0$ for *i* with $y_{i}>0$ and $x_{i\cdot }\gamma^{*}\leq 0$ for *i* with $y_{i}=0$ , see Appendix 1 for a replication of their proof in the special case of Poisson regression. If such a linear combination exists, we say that the data are ‘separated’. From a geometrical point of view, the data are separated if and only if there exists a hyperplane such that all observations with $y_{i}>0$ lie on the plane and all observations with $y_{i}=0$ lie on one side of the plane or also on the plane. For the corona virus testing study, multiplying the dummy variable for supermarket employees by −1 represents a linear combination satisfying the condition given by Correira et al. More generally, we observe that the ML estimate would exist if and only if for each category (supermarket, nursing home, and hospital) at least one person had been tested positive. The existence of the ML estimate only depends on the number of events (people tested positive), but not on the number of people tested in total (Table 1, last column). This observation highlights the difference to the concept of separation in logistic regression (Albert and Anderson, 1984): for the corona virus testing study, ML estimates in logistic regression would exist, if and only if at least one person had been tested positive and at least one person had been tested negative for each category. In the following, we will always use the term separation in the context of Poisson regression as defined above.

As in logistic regression, adding covariates will not remove the nonexistence in Poisson regression. While numerical ML algorithms may declare convergence when the log-likelihood cannot be improved by a further iteration,^14^ ‘a spurious solution is characterized by a “perfect” fit for the observations with $y_{i}=0$ ’,that is, $\exp\;(x_{i\cdot }\hat{\beta })\to 0$ for all $y_{i}=0$ .

### 3.3 Firth’s likelihood penalization applied to the Poisson model

Generally, Firth^8^ suggested adding a penalty term to the log-likelihood of exponential family models that resembles the Jeffreys invariant prior such that the penalized log-likelihood becomes:

(1) $$\ell^{*}(\beta )=\ell (\beta )+(1/2)\log\;|I(\beta )|$$

where |I(β)| denotes the determinant of the Fisher information matrix. The modification is motivated by elimination of bias of order $O(n^{-1})$ in the ML estimates of β , and various empirical studies have proven the bias-preventive properties of Firth’s correction. It also prevents the nonexistence of ML estimates of β and has become the default solution to solve this problem for logistic and Cox regression.^5,15^

Already in Firth’s seminal paper,^8^ an example with Poisson regression was included. However, Firth’s likelihood penalization (FL) for the Poisson model has not been studied any further. FL estimates maximizing (1) can be obtained through iteratively solving the modified score equations:

(2) $$U_{j}^{*}(\beta )=\overset{n}{\underset{i=1}{\sum }}\;x_{ij}(y_{i}+h_{i}/2-\exp\;(x_{i\cdot }\beta +z_{i})),\;\;\;\;\;j=0,\ldots ,k$$

where $h_{i}$ are the diagonals of the ‘hat’ matrix $H=XW^{1/2}(X^{\prime }WX{)}^{-1}XW^{1/2}\;$ , with $W=\mathrm{diag}(\exp\;(x_{i\cdot }\beta +z_{i}))$ . Generally, tr(H)=k+1 and $h_{i}>0$ for all *i*. Equation (2) can been written in a form revealing that FL estimates can be obtained by ML estimation on an augmented data set that consists of the original data in which observed outcomes $y_{i}$ were augmented by $h_{i}/2$ . While we expect that Firth’s correction will correct some of the small-sample bias of the ML estimates also in Poisson regression, it will supply predictions for the counts ${\hat{\mu }}_{i}=\exp\;(x_{i\cdot }\hat{\beta }+z_{i})$ , which are slightly too high because the modified score equation $U_{0}^{*}(\beta )$ implies $\sum_{i=1}^{n}(y_{i}+h_{i}/2)=\sum_{i=1}^{n}{\hat{\mu }}_{i}.$

### 3.4 A modified Firth correction to achieve unbiased prediction

Similarly, in logistic regression with rare events, Firth’s penalization provides predicted probabilities that are on average higher than the observed event rate. To solve this problem, Puhr et al.^7^ suggested two methods, ‘FL with intercept correction’ (FLIC) and ‘FL with added covariate’ (FLAC). Here we explore the performance of these methods in the setting of Poisson regression.

FLIC consists of first obtaining the FL solution and then to correct the intercept parameter by adding a constant δ such that $\sum_{i=1}^{n}y_{i}=\sum_{i=1}^{n}{\hat{\mu }}_{i}$ . This is achieved by using the linear predictors from the FL solution, ${\hat{\eta }}_{i}=\;\sum_{\;j=0}^{k}x_{ij}{\hat{\beta }}_{j}+z_{i}$ as offsets in a second logistic regression that only estimates an intercept $\delta^{\mathrm{FLIC}}$ by ML. The regression coefficients ${\hat{\beta }}_{j},\;j=1,\ldots ,k$ , are left unchanged by FLIC, and the new intercept is given by ${\hat{\beta }}_{0}^{\mathrm{FLIC}}={\hat{\beta }}_{0}+{\hat{\delta }}^{\mathrm{FLIC}}$ .

FLAC starts by estimating the FL solution as well, but this is only done to compute the values of $h_{i}$ . Subsequently, an augmented data set is constructed, adding a pseudo observation to each original observation with event count $h_{i}/2$ , and defining an ‘added covariate’ *G* such that it distinguishes original from pseudo-observations by assuming values of 0 and 1 for them, respectively. The augmented data set with the added covariate is then subjected to ML Poisson regression where an additional coefficient $\gamma^{\mathrm{FLAC}}\;$ corresponding to *G* is estimated. For predictions, *G* is assigned a value of 0. As for FLIC, we have $\sum_{i=1}^{n}y_{i}=\sum_{i=1}^{n}{\hat{\mu }}_{i}$ (see Appendix 1).

While the two approaches generally give different results for logistic regression, it is remarkable that not only the FLIC estimates, but also FLAC estimates of $\beta_{1},\ldots ,\beta_{k}$ coincide with those obtained by FL for Poisson regression, and that ${\hat{\beta }}_{0}^{\mathrm{FLIC}}={\hat{\beta }}_{0}^{\mathrm{FLAC}}$ (see Appendix 1).

Because FLIC and FLAC do not modify the FL regression coefficients, CI for the regression coefficients can be obtained out of the FL model, and in the case of data sparsity should preferably be computed by the PPL method.^5,6^ Here, a (1−α)×100 per cent CI for a parameter $\beta_{j}$ is defined as the set of values $\beta_{j}^{*}$ for which

$$2\left[\ell^{*}({\hat{\beta }}^{\mathrm{FL}})-\underset{\beta_{0},\ldots ,\beta_{\;j-1\;},\beta_{\;j+1},\ldots ,\beta_{k}}{\max}\;\ell^{*}(\left(\beta_{0\;},\ldots ,\beta_{\;j-1,}\beta_{j}^{*},\;\beta_{\;j+1},\;\ldots \;,\beta_{k}\right))\right]\leq \chi_{1}^{2}(1-\alpha )$$

where $\max_{\beta_{0},\ldots ,\beta_{\;j-1\;},\beta_{\;j+1},\ldots ,\beta_{k}}\ell^{*}(\left(\beta_{1\;},\ldots ,\beta_{\;j-1,}\beta_{j}^{*},\;\beta_{\;j+1},\;\ldots \;,\beta_{k}\right))$ is the PPL, that is, the penalized log-likelihood fixed at $\beta_{j}=\beta_{j}^{*}$ and maximized over $\beta_{\;j^{\prime }};{\;j}^{\prime }\in \{0,\ldots ,k\}\setminus j\;$ ; and $\chi_{1}^{2}(1-\alpha )$ is the (1−α) quantile of the chi-squared distribution with one degree of freedom. PPL CI can become asymmetric and in such a case indicate the inadequacy of the Wald method for CI estimation.

We have written a SAS macro FLACPOISSON that performs the Firth correction and the FLAC modification based on iterated data augmentation using repeated calls to PROC GENMOD (https://github.com/georgheinze/flicflac). For simplicity, we approximate the PPL CI in FLACPOISSON by evaluating the profile likelihood (PL) of the augmented data fixing the event counts of the pseudo data at the values $h_{i}/2\;\;$ obtained at the FL solution. In LogXact,^10^ PPL for FL is available where the hat diagonals are iteratively updated when computing the confidence limits. We will illustrate the difference between the two methods in an example of the occurrence of complications in implant dentistry.

### 3.5 Alternative methods do deal with separation

#### 3.5.1 Exact conditional Poisson regression with median unbiased estimation

In exact conditional Poisson regression, inference is based on the exact conditional likelihood of a parameter $\beta_{j}$ conditional on the observed sufficient statistics $t_{{\;j}^{\prime }}$ of all other parameters $\beta_{{\;j}^{\prime }};{\;j}^{\prime }\in \{1,\ldots ,k\}\setminus j$ , where a sufficient statistic $t_{{\;j}^{\prime }}$ is given by $t_{\;j^{\prime }}=\sum_{i=1}^{n}x_{i{\;j}^{\prime }}y_{i}$ .^13^ The maximum conditional likelihood estimate (MCLE) is the value of $\beta_{j}$ that maximizes its exact conditional likelihood. In case a finite MCLE does not exist, it can be replaced by a median unbiased estimate (MUE).^16^ If the exact distribution is degenerate, neither MCLE nor MUE can be computed.

Exact conditional Poisson regression does not provide an estimate of the intercept and thus cannot be used for prediction. The implementations in SAS/PROC GENMOD, in Cytel studio, and in Cytel’s PROC LOGXACT add-on for SAS^10^ allow for computation of exact and mid-p CIs and more details can be found in the software documentation.^17^ In the remainder, we will refer to this method as EP regression.

#### 3.5.2 Bayesian data augmentation

Bayesian estimation with properly specified priors also solves the separation issue. To overcome problems of computing time and diagnostics in Bayesian analysis with Markov chain Monte Carlo algorithms, Sullivan and Greenland^18^ illustrate the use of data augmentation to specify normal priors, including an example for Poisson regression. The spread of the normal prior for a regression coefficient is determined by the width of a prior interval for the associated IRR. For example, if a 95% prior interval for the IRR of (1/1000, 1000) is specified, then based on a normal distribution the prior standard error is log(1000)/1.96=3.52 , suggesting a prior variance *v* of $3.52^{2}=12.39.$ The prior distribution can be specified by adding pseudo-observations, one for each regression coefficient, with a value of 1/*S* for the associated covariate and 0 for all other covariates, where *S* denotes an approximation constant (higher values giving a better approximation). No pseudo-observations are specified for the intercept. The event count of each pseudo observation is set to $y=S^{2}/v$ , and the corresponding offset to z=log(y) . While Sullivan and Greenland^18^ chose S=25 , we used S=10,000 to obtain from the pseudo-observations 95% PL CI for the regression coefficients that were symmetric up to the third decimal place. After data augmentation, maximum posterior estimates can be computed by applying ML methods to the augmented data, and intervals for them from PL. Bayesian data augmentation (BDA) results in unbiased predicted counts in the sense that $\sum_{i=1}^{n}y_{i}=\sum_{i=1}^{n}{\hat{\mu }}_{i}$ .

## 4 A simulation study

### 4.1 Methods

The methodology of our simulation study is described as recommended by Morris et al.^19^

**Aims:** We intended to explore and compare the performance of different estimation methods for Poisson regression with sparse data.

**Data-generating mechanisms**: To capture a plausible context, we considered a data-generating scheme as described by Binder et al.^20^ and Zöller et al.^21^ First, we generated data sets of *n* observations on 10 covariates $X_{1},\ldots ,\;X_{10}$ of different, prespecified distributions that were obtained by applying certain transformations to normal random variables $Z_{1},\ldots ,\;Z_{10}$ sampled from a standard multivariate normal distribution with correlation matrix Σ (Table 2). In this way, we generated four binary covariates $X_{1},\ldots ,\;X_{4}$ , two ordinal covariates $X_{5}$ and $X_{6}$ with three levels, and four continuous covariates $X_{7},\ldots ,\;X_{10}$ . The correlation structure of the variables $Z_{1},\ldots ,Z_{10}$ is transferred to the variables $X_{1},\ldots ,X_{10}$ in a somewhat attenuated way.

**Table 2.** Covariates generated in the simulation study. *I*(*x*) is the indicator function that equals 1 if the argument *x* is true, and 0 otherwise. [*x*] indicates that the non-integer part of the argument *x* is eliminated.

$z_{j}$	**Correlation of** $z_{j}$	**Type**	$x_{j}$	$E(x_{j})$
$z_{1}$	$z_{2}\;(0.5),\;z_{7}\;(0.5)$	Binary	$x_{1}=I(z_{1}>1.28)$	0.1
$z_{2}$	$z_{1}\;(0.5)$	Binary	$x_{2}=I(z_{2}>0.35)$	0.36
$z_{3}$	$z_{4}\;(-0.5),\;z_{5}(-0.3)$	Binary	$x_{3}=I(z_{3}>0)$	0.5
$z_{4}$	$z_{3}(-0.5),\;z_{5}(0.5),\;z_{7}(0.3),\;z_{8}(0.5),\;z_{9}(0.3)$	Binary	$x_{4}=I(z_{4}>0)$	0.5
$z_{5}$	$z_{3}(-0.3),\;z_{4}(0.5),\;z_{8}(0.3),\;z_{9}(0.3)$	Ordinal	$x_{5}=I(z_{5}\geq -1.2)+I(z_{5}\geq 0.75)$	1.11
$z_{6}$	$z_{7}(-0.3),\;z_{8}(0.3)$	Ordinal	$x_{6}=I(z_{6}\geq 0.5)+I(z_{6}\geq 1.5)$	0.37
$z_{7}$	$z_{1}(0.5),\;z_{4}(0.3),\;z_{6}(-0.3)$	Continuous	$x_{7}=[10\cdot z_{7}+55]$	54.5
$z_{8}$	$z_{4}(0.5),\;z_{5}(0.3),\;z_{6}(0.3),\;z_{9}(0.5)$	Continuous	$x_{8}=[\max(0,100\cdot \exp\;(z_{8})-20)]$	131.1
$z_{9}$	$z_{4}(0.3),\;z_{5}(0.3),\;z_{8}(0.5)$	Continuous	$x_{9}=[\max(0,\;80\cdot \exp\;(z_{9})-20)]$	1.77
$z_{10}$		Continuous	$x_{10}=[10\cdot z_{10}+120]$	119.5

To avoid extreme values for the two log-normally distributed covariates $X_{8}$ and $X_{9}\;$ , we used truncated normal distributions (with truncation at the 99th percentile) to generate them. We generated the data sets using the R package simdata.^22^

We considered a full factorial design, varying the number of covariates, k∈{2,5,10} , the events per variable (EPV) ratio, EPV∈{3,5,10} , and the true regression coefficient (log IRR) of $X_{1}$ , $\beta_{1}\in \{-\log\;(16),\;-\log\;(8),-\log\;(4),\;-\log\;(2),\;0,\log\;(2),\log\;(4),\log\;(8),\;\log(16)\}$ . We kept all other $\beta_{j}$ fixed with $\beta_{2},\beta_{4}=0.69;\;\beta_{3}=-0.69;\;\beta_{5}=0.35;\;\beta_{6}=-0.35;\beta_{7},\beta_{9}=0.69/\mathrm{ISR};\beta_{8},\beta_{10}=-0.69/\mathrm{ISR}$ , where ISR was the intersextile range (difference between fifth and first sextile) of the corresponding continuous covariate. The intercept $\beta_{0}$ was chosen such that the marginal event incidence was ∼0.1. We simulated a rate multiplier ψ following our example on the occurrence of complications in implant dentistry (see below) by sampling from a zero-truncated Poisson distribution (restricted to numbers greater than 0) with mean 1.6. The outcome (number of events) $y_{i}\;$ was then drawn from a Poisson distribution with parameter $\mu_{i}=\exp\;(\eta_{i})=\exp\;(\beta_{0}+x_{i1}\beta_{1}+\cdots +x_{ik}\beta_{k})\psi_{i}$ . The sample size was determined by fixing the expected EPV ratio at desired values typical for sparse epidemiological data sets (3, 5, or 10). This resulted in 81 possible combinations of simulation parameters. We simulated 10,000 data sets with each of those combinations.

**Methods**: We analysed each simulated data set by fitting a Poisson regression model including $\log\;\psi_{i}$ as an offset variable and estimating the regression coefficients by maximizing the likelihood (ML), using BDA based on prior intervals for the IRR of (1/1000, 1000) for binary and ordinal covariates and of (1/100, 100) for continuous covariates, using FL, and using FLAC. We also included EP regression in simulated scenarios where it was computationally feasible, that is, with k≤5 and n≤250 .

We estimated 95% CI for regression coefficients by the Wald method for ML using PROC GENMOD, and by likelihood profiles applied to the augmented data for BDA, FL, and FLAC (FLACPOISSON macro). In the case of separation, the values for ML are those reported by PROC GENMOD at the last iteration.

Exact point and interval estimates and mid-p corrected CI were obtained by PROC LOGXACT.^10^

**Estimands**: The estimands in this study were the expected event counts $\mu_{i}$ and the regression coefficient $\beta_{1}$ . We also evaluated the frequency of nonexistence of ML estimates (separation).

**Performance measures**: For point estimates of β and predictions, we evaluated bias and root mean squared error (RMSE) $\;\times \sqrt{n}$ . For predictions, the mean squared prediction error was obtained as $n^{-1}\sum_{i=1}^{n}{\left(\hat{\mu_{i}}-\mu_{i}\right)}^{2}\;$ in each data set and then averaged over all simulated data sets in a scenario. The root of the averaged mean squared prediction error (root mean squared prediction error (RMSPE)) times $\sqrt{n}$ is reported. For CI of β , we evaluated left/right-tailed one-sided coverage rates (nominal levels 0.975) and power (probability to exclude 0). We summarized the simulation results graphically using nested loop plots.^23,24^

### 4.2 Results

#### 4.2.1 Incidence of separation

The incidence of separation was generally higher in scenarios with smaller sample sizes and with larger negative values of $\beta_{1}$ , see Figure S1. The latter phenomenon is a consequence from the imbalance of $X_{1}$ , where with negative $\beta_{1}$ events were less likely in the less frequent group ( $X_{1}=1$ ), and data sets with no events when $X_{1}=1$ occurred more frequently. Among the scenarios with a given EPV ratio and a given value of $\beta_{1}$ , scenarios with 10 covariates often had the fewest separated data sets. This might seem counterintuitive since, for a fixed non-separated data set, omitting covariates can never induce separation. However, in our simulation study, scenarios with 2, 5, and 10 covariates do not only differ in the number of covariates but also in the type of covariates, the magnitude of the intercept, and the sample size.

We only included EP in the comparison of methods for simulation scenarios with n≤250 and k≤5 . For larger data sets, its application was computationally not feasible or not possible because with continuous covariates (when k=10 ) degenerate distributions of sufficient statistics were encountered for which no inference is possible. The MCLE in EP did not exist and had to be replaced by the MUE for almost the same data sets where ML estimation failed, see Figure S1. Only with large positive values of $\beta_{1}$ and small-sample sizes, there were considerably more data sets where the MCLE had to be replaced by the MUE than separated data sets. Finally, there were a few datasets where neither the MCLE nor the MUE existed (at most 0.7% of data sets, as observed for the scenarios with n=60 , k=2 and $\beta_{1}=-\log(16)$ or $\beta_{1}=-\log(4)$ ).

#### 4.2.2 Predictions

**Figure 1.**
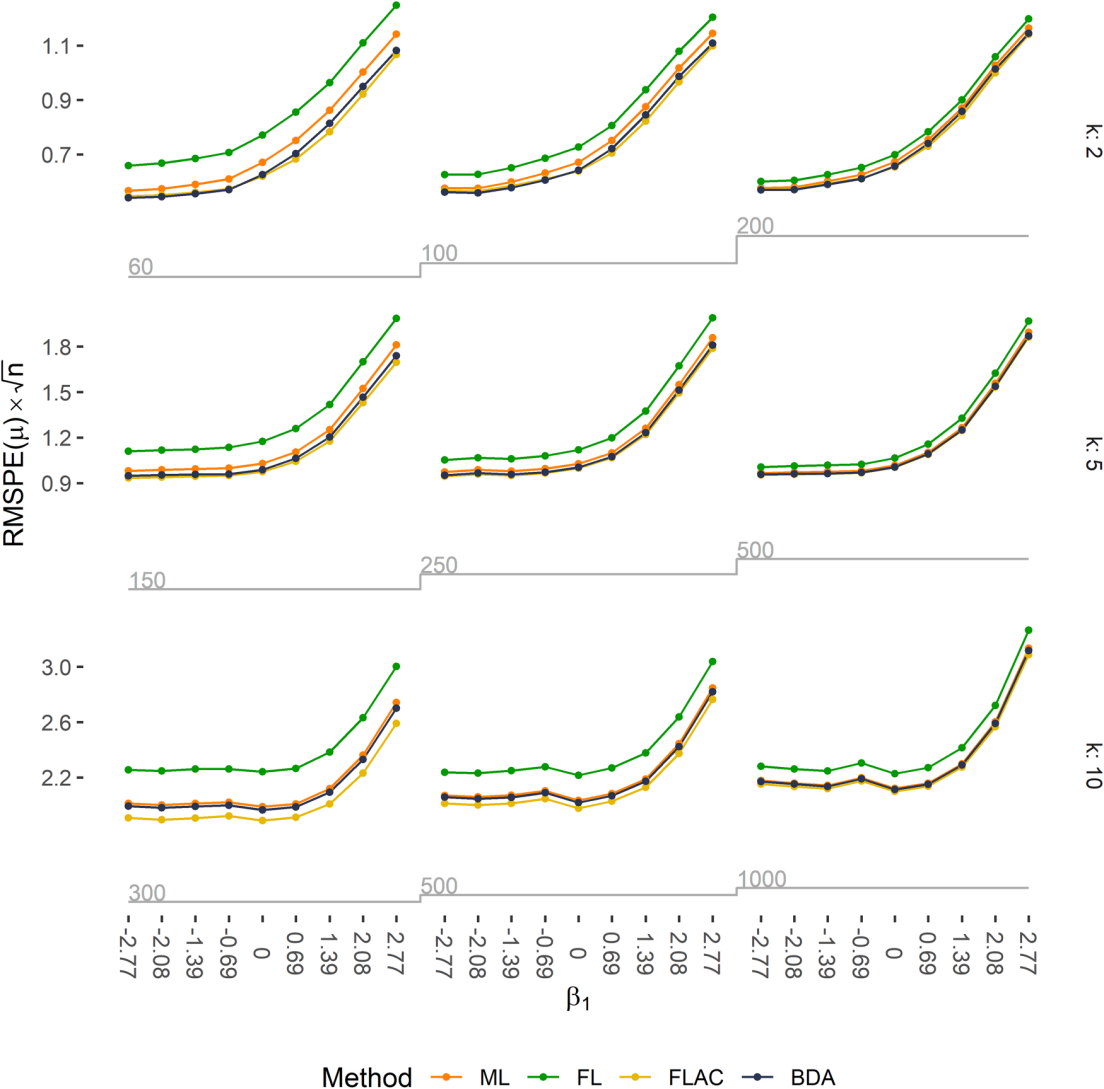
Predictive accuracy is expressed as RMSPE(μ) multiplied by the square root of the sample size *n* for all 81 simulation scenarios. Expected counts were obtained by ML, FL, FLAC, and BDA. Rows correspond to the number of covariates in the respective simulation scenario, columns to the EPV ratio, and ticks on the *x*-axis to the true value of $\beta_{1}$ . Grey step functions below the plots indicate the sample size.

RMSPE: root mean squared prediction error; ML: maximum likelihood; FL: Firth’s likelihood penalization; FLAC: FL with added covariate; BDA: Bayesian data augmentation; EPV: events per variable.

The description of the prediction performance is restricted to the methods ML, FL, FLAC, and BDA, since XL does not allow for predictions. Across all methods, predictions were more accurate in terms of RMSPE( μ ) for simulation scenarios with fewer variables or with higher EPV ratio. Throughout all evaluated scenarios, FLAC and BDA yielded the most accurate predictions, followed by ML and FL, which, because of the overprediction, performed worst, see Figure 1. With FL, the bias in predictions is fully characterized by the number of covariates, in fact the sum of predictions $\sum_{i=1}^{n}{\hat{\mu }}_{i}$ overestimates the sum of observed counts $\sum_{i=1}^{n}y_{i}$ by (k+1)/2 . As described in Section 3, ML, FLAC, and BDA yield unbiased predictions in the sense that $\sum_{i=1}^{n}y_{i}=\sum_{i=1}^{n}{\hat{\mu }}_{i}$ . Figure S2 shows the scaled bias and RMSPE(μ) in relation to the true incidence exemplarily for one simulation scenario.

#### 4.2.3 Regression coefficients: point estimates

**Figure 2.**
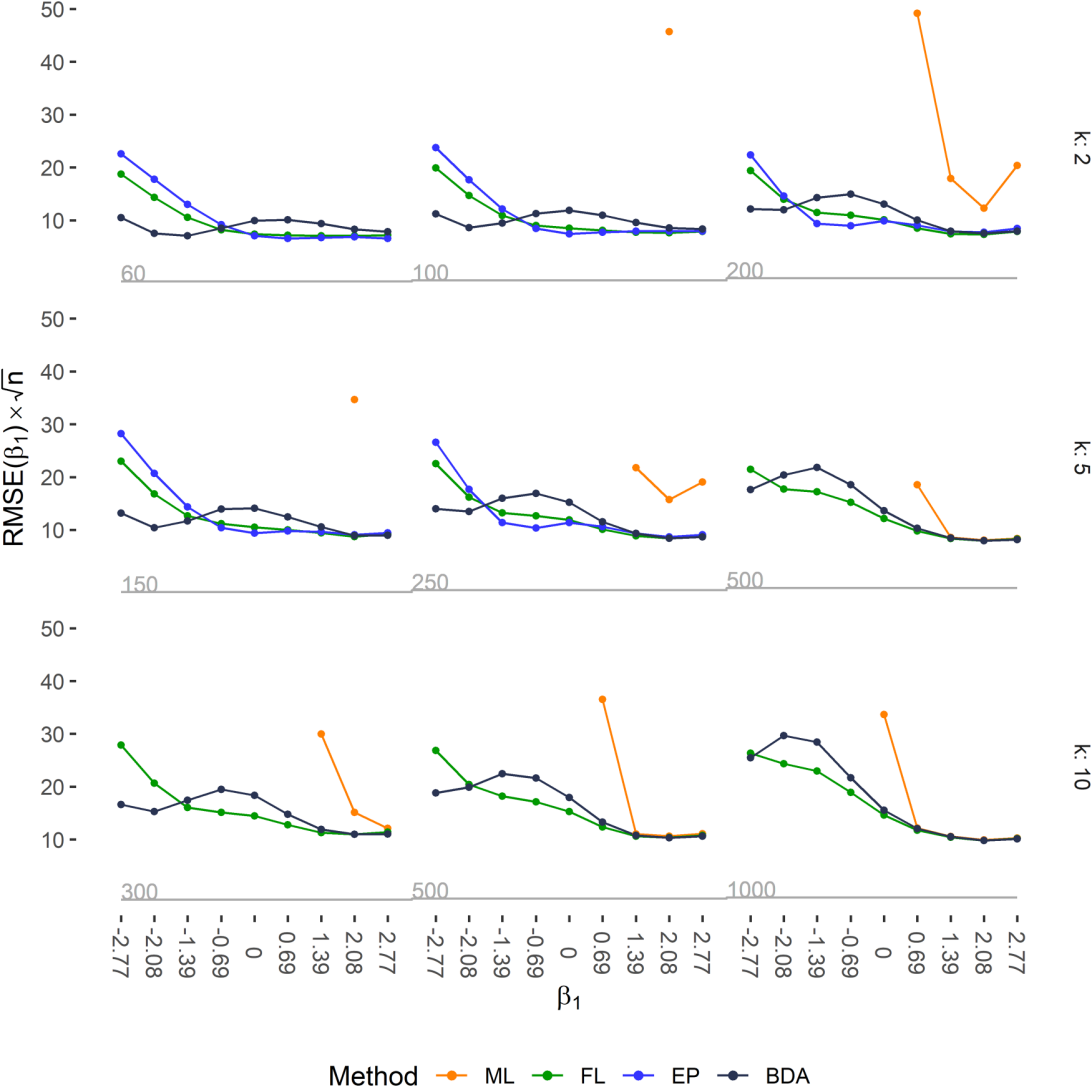
Accuracy of the estimated regression coefficient $\beta_{1}$ , evaluated as RMSE( $\beta_{1}$ ) multiplied by the square root of the sample size *n* for all 81 simulation scenarios coefficients. The regression coefficient $\beta_{1}$ was estimated by ML, FL, EP, and by BDA. Rows correspond to the number of covariates in the respective simulation scenario (*k*), columns to sample size (see numbers in grey), and ticks on the *x*-axis to the true value of $\beta_{1}$ . Grey step functions below the plots indicate sample size. RMSE( $\beta_{1}$ ) for ML occasionally exceeded the upper limit of the plotting range and was then omitted.

RMSE: root mean squared error; ML: maximum likelihood; FL: Firth’s likelihood penalization; EP: exact Poisson; BDA: Bayesian data augmentation.

When $\beta_{1}$ was evaluated as estimand, FLAC was not considered separately as it yields the same regression coefficients as FL. Concerning accuracy of regression coefficients, FL, EP and BDA performed similarly well, with some advantage of BDA for extreme, negative values of $\beta_{1}$ and some advantage of FL and EP for $\beta_{1}$ close to 0, see Figure 2. A major drawback of EP is that it is not applicable with larger sample sizes. The worse performance of ML is partly due to the occurrence of separation, but also to a generally higher inaccuracy for data with a low EPV ratio.

ML lead to a large negative bias because of divergent estimation caused by separation when $\beta_{1}$ was negative, see Figure S3. By contrast, FL, EP and, to a lesser extent, BDA lead to a positive bias in scenarios with large negative values of $\beta_{1}$ , meaning that some bias toward 0 was introduced. The behaviour of the methods was similar in estimating $\beta_{2}$ , see Figures S4 and S5.

#### 4.2.4 Regression coefficients: CIs

**Figure 3.**
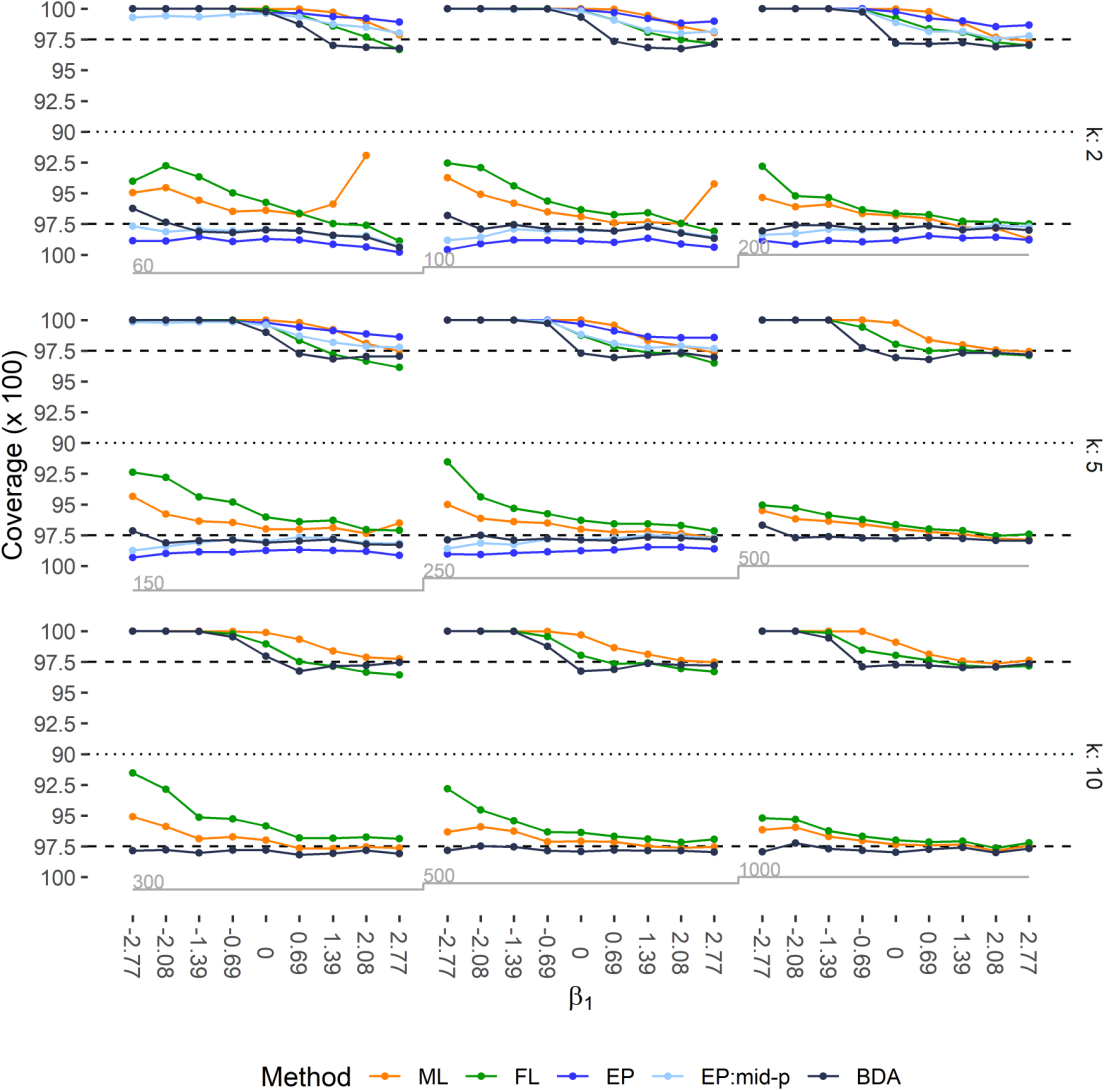
Left-tailed and right-tailed coverage of 95% two-sided CIs for regression coefficient $\beta_{1}$ for all 81 simulation scenarios. CIs were estimated using the Wald method with ML estimation, likelihood profiles with FL, exact interval estimates (EP), and mid-p corrected CIs (EP:Mid-p) with EP regression and likelihood profiles with BDA. The upper halves of the plots describe the right-tailed coverage, lower halves the left-tailed coverage. Nominal levels of 97.5% are marked by dashed lines. Rows correspond to the number of covariates in the respective simulation scenario, columns to the EPV ratio, and ticks on the *x*-axis to the true value of $\beta_{1}$ . Grey step functions below the plots indicate sample size. For one scenario ( n=60 , k=2, $\beta_{1}=\log(16)$ ) the left-tailed coverage of the CIs for ML exceeded the lower limit of the plotting range.

CI: confidence interval; ML: maximum likelihood; BDA: Bayesian data augmentation; EP: exact Poisson; FL: Firth’s likelihood penalization; EPV: events per variable.

Left-tailed and right-tailed coverage rates of 95% two-sided CIs are depicted in Figure 3, and the power to exclude $\beta_{1}=0$ is shown in Figure S6. For large negative $\beta_{1}$ , all methods yielded higher than nominal right-tailed coverage often in combination with low power. In these scenarios ML–Wald intervals showed undercoverage of their left tails despite the considerable amount of separated data sets (cf. Figure S1). This left-tailed undercoverage for large negative $\beta_{1}$ was even more severe with FL–PPL intervals. While exact CIs were over conservative, mid-p intervals could correct the conservatism, resulting in increased power and coverage close to the nominal one-sided level of 97.5%. BDA–PL intervals generally were preferable, both in terms of coverage and power. Median width of CIs is described in Figure S7.

## 5 Implant dentistry study

Feher et al.^25^ studied risk factors for complications in implant dentistry. Risk factors were assessed in 1133 patients undergoing 2405 implantations. We used Poisson regression to model the number of haematological complications by the risk factors age (in decades), smoking (no smoking, light smoking, and heavy smoking), and diabetes mellitus and considered the number of implantations performed per patient as the rate multiplier. Smoking was coded using ‘ordinal coding’, that is, two dummy variables were defined contrasting light smokers from non-smokers, and heavy smokers from light smokers. Table S1 contains the basic descriptives for these variables.

ML analysis failed to converge because no haematological complications were observed for light smokers (Table 3). This caused the regression coefficients of the two dummy variables associated with smoking to diverge. PROC GENMOD reported arbitrarily large estimates and standard errors for the corresponding regression coefficients but rather than reflecting the large standard errors, the reported Wald CI was collapsed at the point estimates.

**Table 3.** Results for implant dentistry study: point estimates of IRRs and 95% CI.

	Age (per decade)	Light versus no smoking	Heavy versus light smoking	Diabetes versus no diabetes
ML, IRR	1.657	0^*^	20.3 × 10^9^^*^	6.012
Wald 95% CI	1.298, 2.116			2.895, 12.486
FL/FLAC, IRR	1.646	0.147	9.045	6.154
PL 95% CI	1.309, 2.101	0.001, 1.047	0.950, 1110.5	2.944, 12.231
PPL 95% CI	1.3, 2.084	0.001, 1.065	0.928, 1209.8	2.901, 12.384
BDA 100/1000, IRR	1.665	0.113	10.339	5.977
PL 95% CI	1.315, 2.143	0.003, 0.867	1.020, 373.4	2.792, 12.105
BDA 5/50, IRR	1.656	0.24	4.686	5.860
PL 95% CI	1.317, 2.130	0.029, 1.055	0.793, 41.133	2.754, 11.788
EP, IRR	1.657	0.213	5.160	6.027
Exact 95% CI	1.296, 2.147	0, 1.217	0.627, ∞	2.630, 13.058
Mid-p 95% CI	1.305, 2.130	0, 0.972	0.856, ∞	2.806, 12.395

ML: maximum likelihood; FL: Firth’s likelihood penalization; FLAC: FL with added covariate; BDA: Bayesian data augmentation; 100/1000 and 5/50: upper prior limit for IRR for age/binary covariates; EP: exact Poisson; PL: profile likelihood; PPL: profile penalized likelihood; CI: confidence interval; IRR: incidence rate ratio.

^*^ Not converged; Wald CIs reported by PROC GENMOD collapsed at point estimate.

By contrast, FL, BDA, and EP using the MUE gave plausible estimates for all variables (Table 3). These methods also supplied 95% CI, which provided some evidence that light smokers experienced fewer haematological complications than non-smokers or heavy smokers. When comparing the two methods of estimating CI for FL, as expected, fixing the pseudo data with weights computed at the maximum penalized likelihood estimate (‘PL CI’ from the augmented data, used in the simulation study) led to slightly narrower intervals than iterating the weights (‘PPL CI’). We also compared the impact of specifying different priors with BDA. Compared to weakly informative priors (‘BDA 100/1000’: 95% prior intervals for the IRR extending to 100 for age and to 1000 for the binary covariates, used in the simulation study), with narrower priors (‘BDA 5/50’: 95% prior intervals extending to 5 for age and to 50 for the binary covariates), all point estimates and nearly all CI were pulled towards unity. The effect of changing the prior was particularly strong for the two IRRs corresponding to smoking which caused the separation problem. Employing weakly informative priors resulted in CI supporting the hypothesis that light smokers experienced fewer complications than non-smokers and heavy smokers, while with narrower priors the CI included unity. EP generally resulted in IRR and CI closer to the estimates by BDA 5/50 than to the estimates by other methods.

While 37 hematologic complications were observed, 39.5 complications were predicted by FL, which estimated the intercept at −4.3728. Applying FLAC changed the intercept to −4.4382, and recalibrated the total number of predicted complications to the observed number of 37. Because age was centred at 50 years, $\exp(\beta_{0})$ expresses the risk of a complication with one implantation for a 50-year-old non-smoking non-diabetic person. This risk was estimated at 1.26% by FL and at 1.18% by FLAC, and for a 70-year-old diabetic at 21.0% or 19.7%, respectively.

## 6 Discussion

We proposed and investigated Poisson regression methods to deal with the problem of separation, which leads to nonexistence of ML estimates. We adapted two modifications of FL, namely FLIC and FLAC, which were originally developed to debias predictions in logistic regression, for Poisson regression. It turned out that in Poisson regression FLIC and FLAC lead to the same estimation method. This method, which we refer to as FLAC, competed well with alternative approaches such as EP regression, which needs special software and is only applicable for small-sized problems, or BDA, which is easy to implement but crucially depends on the choice of the width of the prior distribution. A possible advantage of BDA could arise if a model with many covariates should be fitted. It is essentially equal to ridge regression, and unlike FL can handle situations where the number of covariates exceeds the number of events by regularizing parameter estimates. In our application and simulations we fixed the variance of the prior distribution, which is inversely proportional to the penalty parameter in ridge regression. Optimizing that penalty parameter by, for example, cross-validation invalidates inference about regression coefficients, is not robust to separation^14^ and can lead to instable results.^26^ To sum up, for data sets fitting into the framework of this study, that is, with an EPV ratio of 3 or higher and moderate correlation between covariates, we advocate using FL as it does not need any user input or optimization of a penalty parameter and is computationally feasible, while showing good performance.

In some situations, count outcomes can be naturally reinterpreted as dichotomous outcomes, for example, in the coronavirus testing study where we can either count the number of infections per working place or determine whether a person is infected or not. These data allow for analysis via Poisson regression as well as via logistic regression. It was not the aim of this study to provide guidance on which analysis method to prefer with sparse data, but primarily the decision should depend on the estimand of interest, that is, whether risk ratios or odds ratios should be estimated. Our study employed a realistic design for simulations that resembled data one could typically see in epidemiological studies. Such a design is suitable to draw conclusions on the relative performance of methods in practically relevant situations. While in the simulation study we used R for data generation and summarizing results, we focused on the SAS software for fitting the Poisson models. SAS is widely used among epidemiologists, and with the Cytel SAS procedures, robust and efficient software was available to include EP regression in our comparisons. Hence we employed Cytel’s PROC LOGXACT for EP regression even if an implementation of EP regression is readily available in PROC GENMOD. We provide a SAS macro to apply the Firth correction and its modification FLAC in Poisson regression. The median unbiased estimator implemented in PROC LOGXACT has been described to suffer from extreme shrinkage in case the exact conditional distribution of the sufficient statistic is nearly degenerate. Therefore, an alternative estimator based on maximizing the conditional penalized likelihood was proposed.^27^ We did not include it in our comparison as Heinze and Puhr^27^ found it to be very similar to the FL estimator. We also did not consider the median bias-corrected estimator of Kosmidis et al.^28^

R code for fitting a Poisson model with FL is also available,^29^ however, without the possibility to invoke the FLAC extension or the estimation of PPL CI. We are also not aware of software packages in R which allow fitting EP regression models. Our SAS macro FLACPOISSON, a SAS macro to implement BDA and further SAS macros implementing FL and FLAC for logistic, conditional logistic and Cox regression are available on the GitHub repository https://github.com/georgheinze/flicflac. A further public repository, https://github.com/georgheinze/PoissonF, contains the aggregated data set of the implant dentistry study and code to reproduce its analysis, all codes used to conduct the simulation study, and an R markdown file which summarizes its results.

The FLAC method lends itself to several extensions. Most naturally, it can easily accommodate overdispersion by including the estimation of a dispersion parameter.^30^ This can already be achieved with our SAS macro which is based on PROC GENMOD. Further work could be done to investigate the advantages of considering FLAC for this and other extensions, such as zero-inflated Poisson models and Poisson hurdle models.

## Supplemental Material

sj-docx-1-smm-10.1177_09622802211065405 - Supplemental material for Solutions to problems of nonexistence of parameter estimates and sparse data bias in Poisson regression Supplemental material, sj-docx-1-smm-10.1177_09622802211065405 for Solutions to problems of nonexistence of parameter estimates and sparse data bias in Poisson regression by Ashwini Joshi, Angelika Geroldinger, Lena Jiricka, Pralay Senchaudhuri, Christopher Corcoran and Georg Heinze in Statistical Methods in Medical Research

## Appendix 1

### A.1 Conditions for the existence of ML estimates in Poisson regression

Correia et al.^3^ provide conditions governing the existence of ML estimates for a wide class of generalized linear models. Here, we apply their proof to the special case of Poisson models. We use the notation from the main text.

Theorem 1. Let the n×(k+1) design matrix *X* be of full rank. ML estimation does not give a finite solution for the Poisson regression model if and only if there exists a non-zero vector $\gamma^{*}\in R^{k+1}$ with

$$\begin{array}{rl} & x_{i\cdot }\gamma^{*}=0\;\;\;\;\mathrm{for}\;i\;\mathrm{with}\;y_{i}>0\;\mathrm{and}\\ & \begin{array}{c} x_{i\cdot }\gamma^{*}\leq 0\;\;\;\;\mathrm{for}\;i\;\mathrm{with}\;y_{i}=0. \end{array} \end{array}$$

Proof. The log-likelihood is given by $\ell (\beta )=\sum_{i=1}^{n}-\exp\;(x_{i\cdot }\beta +z_{i})+(x_{i\cdot }\beta +z_{i})y_{i}-\log\;(y_{i}!)$ . Let $\beta ,\gamma \in R^{k+1}$ be arbitrary vectors with γ≠0 and let k>0 be a positive scalar. Differentiating ℓ(β+kγ) with respect to *k* yields

$$\frac{\partial \ell (\beta +k\gamma )}{\partial k}=\overset{n}{\underset{i=1}{\sum }}-\exp\;(x_{i\cdot }\beta +x_{i\cdot }k\gamma +z_{i})x_{i\cdot }\gamma +x_{i\cdot }\gamma y_{i}$$

We will first show that if there exists $\gamma^{*}$ fulfilling properties A1, then the log-likelihood function ℓ(β) is always increasing in the direction of $\gamma^{*}$ , in other words there does not exist a finite β maximizing the log-likelihood. For $\gamma^{*}$ satisfying conditions A1, the directional derivative reduces to

$$\frac{\partial \ell (\beta +k\gamma^{*})}{\partial k}=\underset{i:y_{i}=0}{\sum }-\exp\;(x_{i\cdot }\beta +x_{i\cdot }k\gamma +z_{i})x_{i\cdot }\gamma$$

This expression is greater than 0 since the exponential function is positive and $x_{i\cdot }\gamma *\leq 0$ for all *i* with $y_{i}=0$ and $x_{i\cdot }\gamma^{*}<0$ for some *i* with $y_{i}=0$ . (The inequality $x_{i}\gamma^{*}\leq 0$ has to be strict for at least one observation because otherwise the full rank assumption would be violated.) This proves that for any β and for any k>0 we have $\ell (\beta +k\gamma^{*})>\ell (\beta )$ , that is, we cannot find an ML solution maximizing ℓ . Next, we will show that if there does not exist $\gamma^{*}$ with the properties A1, then the log-likelihood has a maximum. Assume that there does not exist $\gamma^{*}$ satisfying A1. Then for every $\gamma \in R^{k+1}$ at least one of the following two conditions must be true:

1. there is *j* such that $x_{j}\gamma \neq 0$ and $y_{j}>0$ ,
2. there is *j* such that $x_{j}\gamma >0$ and $y_{j}=0$ .

Denote by $\ell_{i}(\beta )=-\exp\;(x_{i\cdot }\beta +z_{i})+(x_{i\cdot }\beta +z_{i})y_{i}-\log\;(y_{i}!)$ the summand in the log-likelihood function corresponding to the *i*th observation. One can show that if any of the two conditions above holds, then we have $\lim_{k\to \infty }\ell_{j}(\beta +k\gamma )=\;-\infty$ . Since the components $\ell_{i}(\beta )$ have a common upper bound we can find a positive scalar $\bar{k}$ such that $\ell (\beta +k\gamma )=\underset{i}{\sum }\;\ell_{i}(\beta +k\gamma )<\underset{i}{\sum }\;\ell_{i}(\beta )=\ell (\beta )$ for any $\beta ,\;\gamma \in R^{k+1}$ and $k>\bar{k}$ . This means that following the log-likelihood from any starting point β in any direction γ will finally yield a decrease in the log-likelihood, guaranteeing the existence of a finite ML solution. □

### A.2 Properties of FLAC

#### A.2.1 The sum of the predicted counts with FLAC, $\sum_{i}{\hat{\mu }}_{i}$ , is equal to $\sum_{i}y_{i}$

With FLAC, the score equation $U_{0}^{\mathrm{FLAC}}$ corresponding to the intercept reads

$$U_{0}^{\mathrm{FLAC}}(\beta ,\gamma )=\underset{i}{\sum }\;(y_{i}-\exp\;(x_{i\cdot }\beta +z_{i}))+\underset{i}{\sum }\;(h_{i}/2-\exp\;(x_{i\cdot }\beta +z_{i}+\gamma \;))=0$$

and the score equation $U_{k+1}^{\mathrm{FLAC}}$ corresponding to the additional covariate γ reads

$$U_{k+1}^{\mathrm{FLAC}}(\beta ,\gamma )=\underset{i}{\sum }\;(h_{i}/2-\exp\;(x_{i\cdot }\beta +z_{i}+\gamma \;))=0.$$

Combining the two equations, we conclude that $\sum_{i}y_{i}=\sum_{i}\exp\;(x_{i\cdot }\beta +z_{i})=\sum_{i}{\hat{\mu }}_{i}$ .

#### A.2.2 FLAC estimates of $\beta_{1},\ldots \;,\beta_{k}$ agree with the FL estimates in Poisson regression

Consider the score equations, $U_{0}^{\mathrm{FL}},\ldots ,U_{k}^{\mathrm{FL}}$ , of FL given as follows:

(A1)
$$\begin{array}{rl} U_{j}^{\mathrm{FL}}(\beta )= & \underset{i}{\sum }\;x_{ij}(y_{i}+h_{i}/2-\exp\;(x_{i\cdot }\beta +z_{i}))\\ = & \;\underset{i}{\sum }\;x_{ij}y_{i}+\underset{i}{\sum }\;x_{ij}h_{i}/2-\exp\;(\beta_{0})\underset{i}{\sum }\;x_{ij}\exp\;(x_{i1}\beta_{1}+\ldots +x_{ik}\beta_{k}+z_{i})=0 \end{array}$$

The corresponding equations for FLAC are given by:

(A2)
$$\begin{array}{rl} U_{j}^{\mathrm{FLAC}}(\beta ,\gamma )= & \underset{i}{\sum }\;x_{ij}(y_{i}-\exp\;(x_{i\cdot }\beta +z_{i}))+\underset{i}{\sum }\;x_{ij}(h_{i}/2-\exp\;(x_{i\cdot }\beta +z_{i}+\gamma \;))\\ = & \underset{i}{\sum }\;x_{ij}y_{i}+\underset{i}{\sum }\;x_{ij}h_{i}/2-(\exp\;(\beta_{0})+\exp(\beta_{0}+\gamma ))\underset{i}{\sum }\;x_{ij}\exp\;(x_{i1}\beta_{1}+\cdots +x_{ik}\beta_{k}+z_{i})=0. \end{array}$$

This shows that estimates of $\beta_{1},\ldots \;,\beta_{k}$ by FL and FLAC are identical.

#### A.2.3 FLIC and FLAC give identical estimates $\beta_{0},\ldots ,\beta_{k}$ in Poisson regression

We have shown above that FLAC estimates of $\beta_{1},\ldots ,\beta_{k}$ agree with the FL estimates. The same is true for FLIC by definition. Moreover, for both methods the sum of the predicted counts $\sum_{i}{\hat{\mu }}_{i}$ is equal to $\sum_{i}y_{i}.\;$ This implies that FLIC and FLAC also give identical estimates for the intercept $\beta_{0}$ .
