# Supplementary material for: Solutions to problems of nonexistence of parameter estimates and sparse data bias in Poisson regression
Source: Stat Methods Med Res. 2021 Dec 21;31(2):253–66. doi: 10.1177/09622802211065405 (PMC8829730; doi:10.1177/09622802211065405)
Supplement: sj-docx-1-smm-10.1177_09622802211065405 - Supplemental material for Solutions to problems of nonexistence of parameter estimates and sparse data bias in Poisson regression [file sj-docx-1-smm-10.1177_09622802211065405.docx]

Supplementary material for

# A solution to problems of nonexistence of parameter estimates and sparse data bias in Poisson regression

by Ashwini Joshi, Angelika Geroldinger, Lena Jiricka, Pralay Senchaudhuri, Christopher Corcoran, Georg Heinze

Figure S1: Relative frequency of simulated data sets with convergence problems in maximum likelihood (ML) estimation or in exact Poisson regression (EP) for all 81 simulation scenarios. The yellow line describes the relative frequency of simulated data sets where the ML estimate for the regression coefficient $\beta_{1}$ did not exist. The blue line describes the frequency of data sets where the maximum conditional likelihood estimate (MCLE) for $\beta_{1}$did not exist and was replaced by the median unbiased estimate (MUE). Rows correspond to the number of covariates in the respective simulation scenario, columns to the events per variable ratio and ticks on the x-axis to the true value of $\beta_{1}$. Grey step functions below the plots indicate sample size.


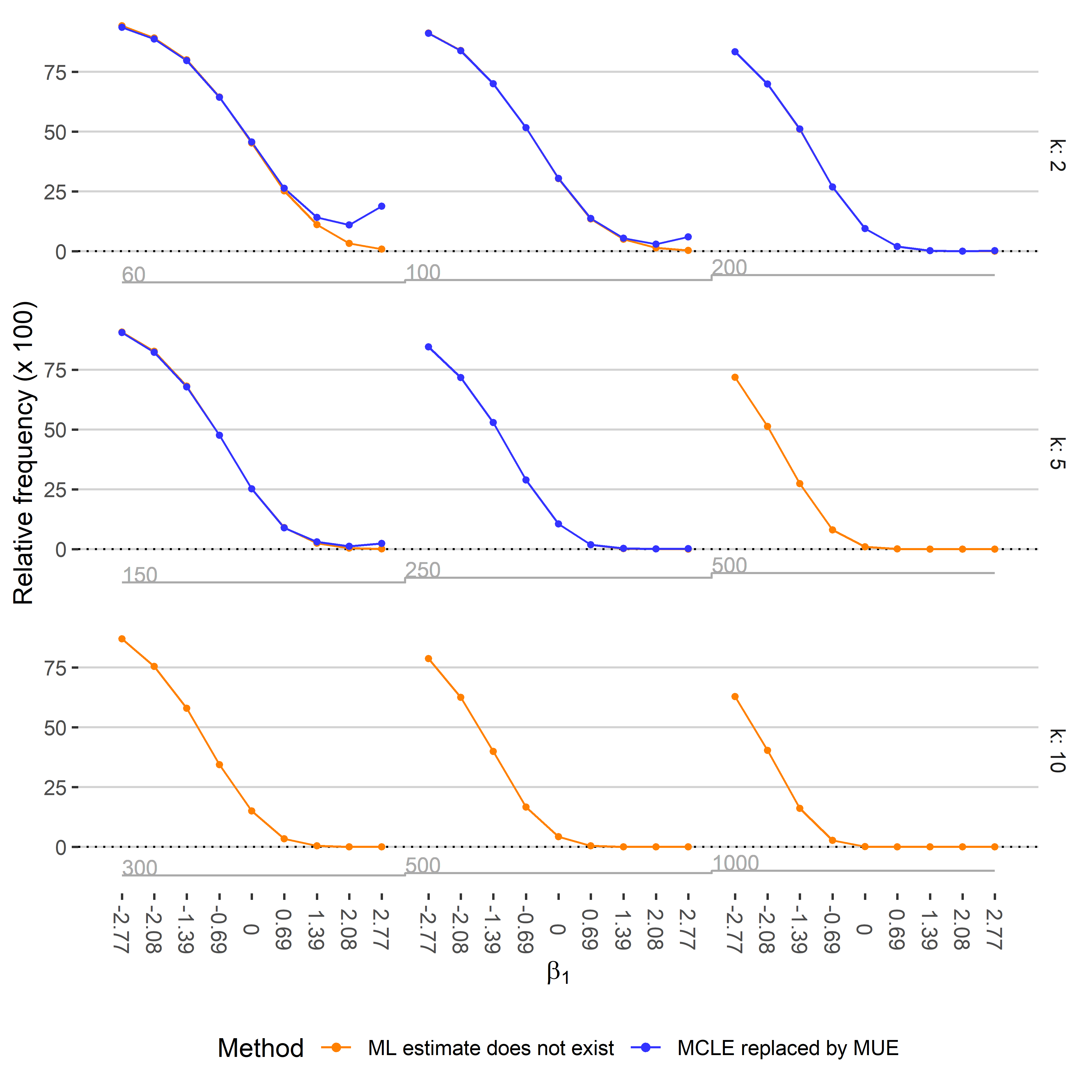


Figure S2: Bias of predictions and root mean squared prediction error (RMSPE) scaled by the standard error of the Poisson parameter, $\sqrt{\mu}$, with $\mu$ the true incidence as shown on the x-axis, exemplified for the scenario with 10 predictors, $\beta_{1}=-log(16)$ and sample size of 300 in the upper row and for the scenario with 10 predictors, $\beta_{1}=log(4)$ and sample size of 300 in the lower row. For the calculation of the bias and RMSPE at various true incidences $\mu$, data were binned into 30 equally-sized groups. Grey vertical lines mark the deciles of the true incidence.


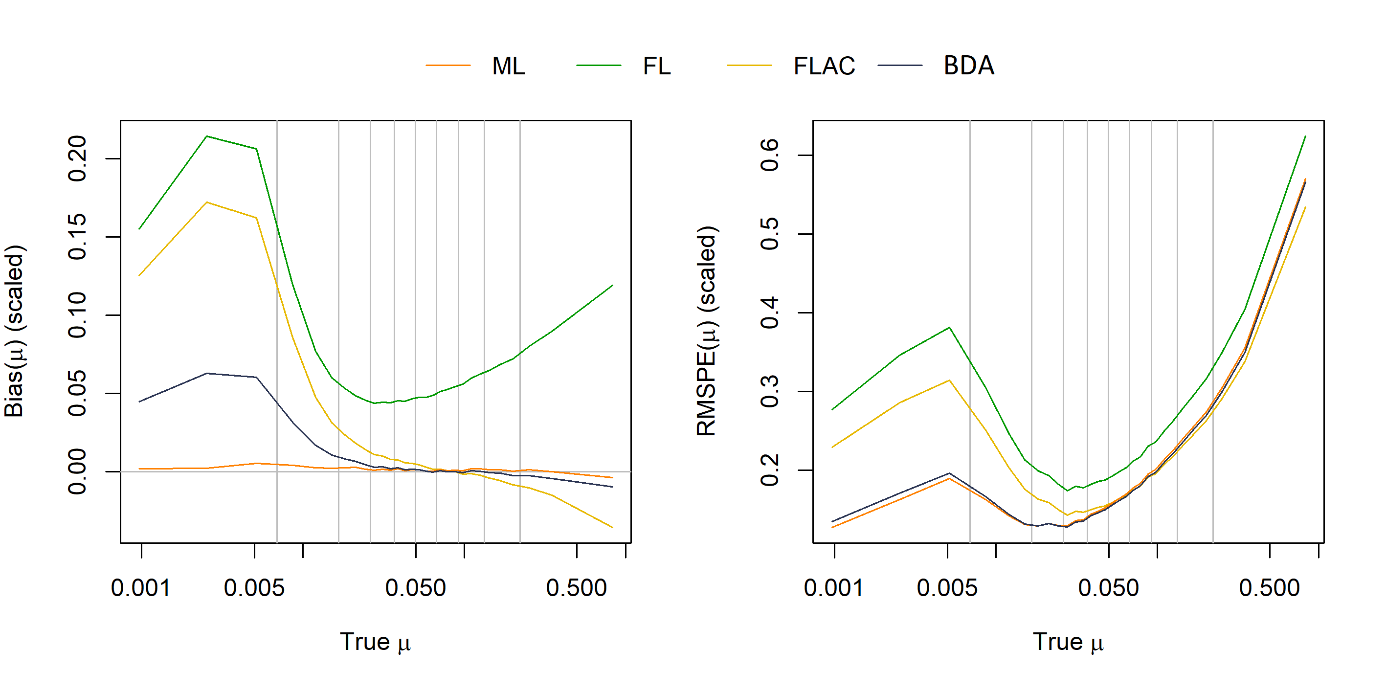


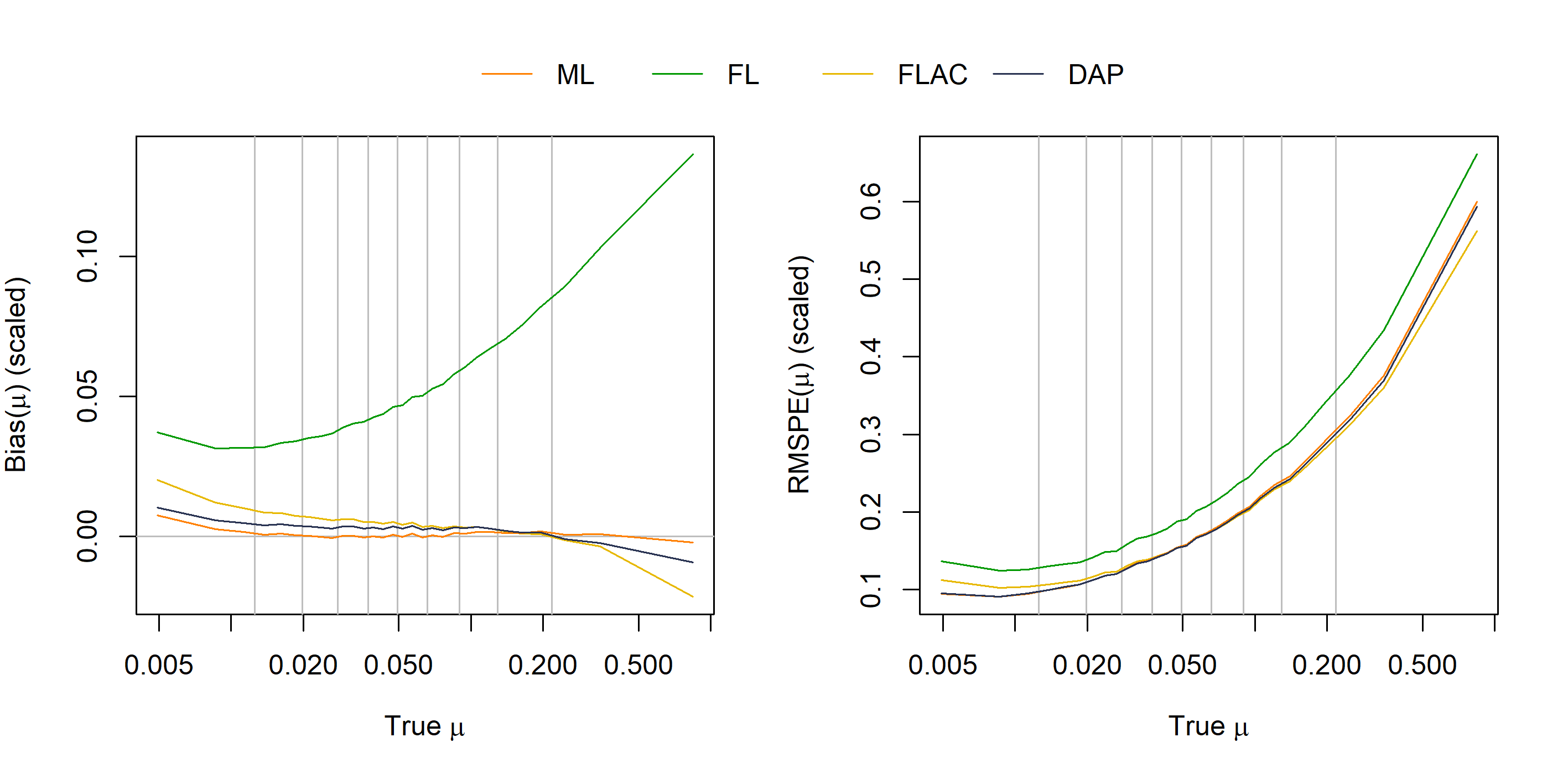


Figure S3: Bias of regression coefficient $\beta_{1}$ for all 81 simulation scenarios. Regression coefficient $\beta_{1}$ was estimated by maximizing the likelihood (ML), by maximizing Firth’s penalized likelihood (FL), by exact Poisson regression (EP) and by using Bayesian data augmentation (BDA). Rows correspond to the number of covariates in the respective simulation scenario, columns to the events per variable ratio and ticks on the x-axis to the true value of $\beta_{1}$. Grey step functions below the plots indicate sample size. The bias for ML is smaller than the lower limit of the plotting range if not shown in the Figure.


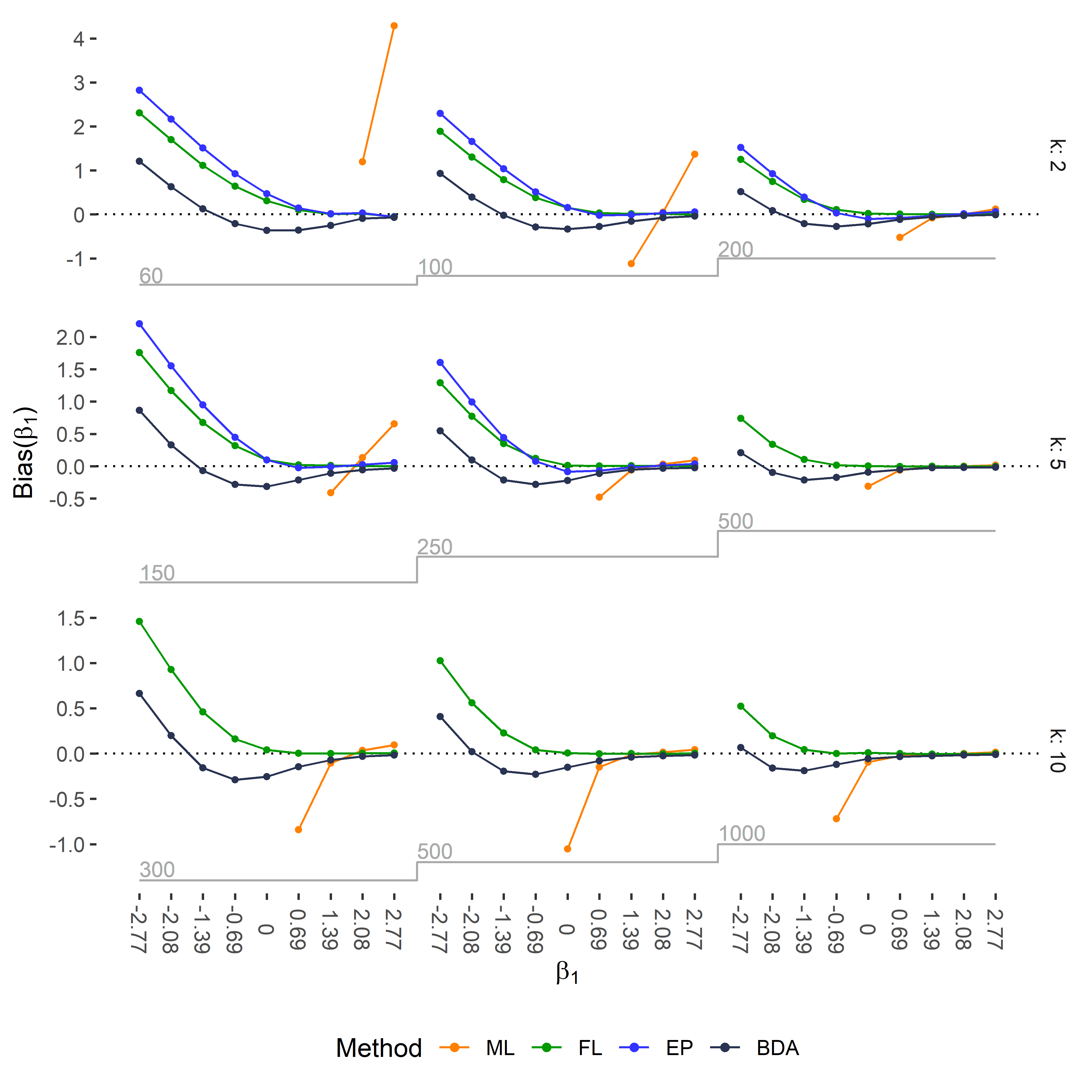


Figure S4. Accuracy of estimated regression coefficient $\beta_{2}$, evaluated as root mean squared error (RMSE($\beta_{2}$)) multiplied by the square root of the sample size $n$ for all 81 simulation scenarios coefficients. Regression coefficient $\beta_{2}$ was estimated by maximizing the likelihood (ML), by maximizing Firth’s penalized likelihood (FL), by exact Poisson regression (EP) and by Bayesian data augmentation (BDA). Rows correspond to the number of covariates in the respective simulation scenario (k), columns to sample size (see numbers in gray) and ticks on the x-axis to the true value of $\beta_{1}$. Grey step functions below the plots indicate sample size. The RMSE($\beta_{2}$) for ML is larger than the upper limit of the plotting range if not shown in the Figure.


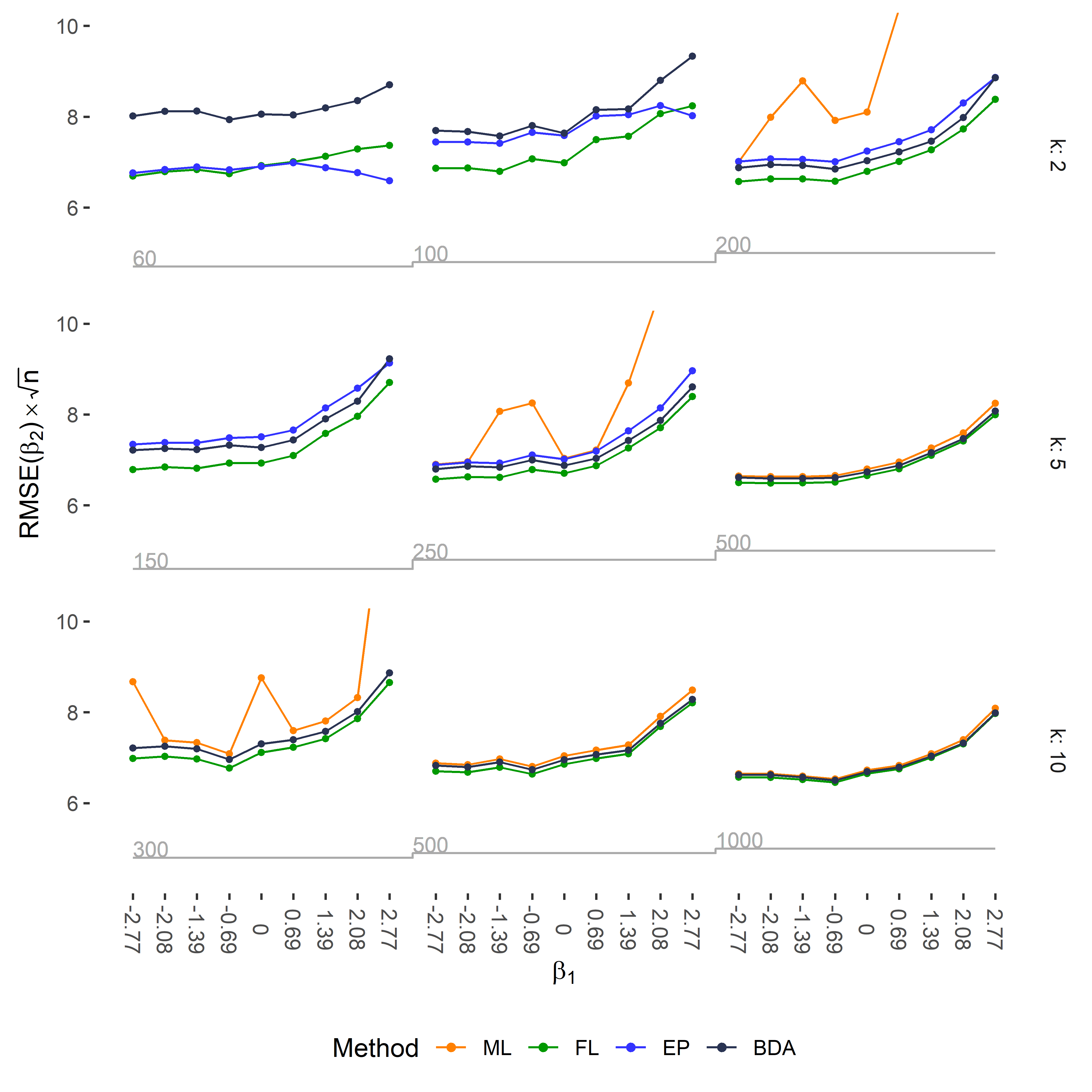


Figure S5: Bias of regression coefficient $\beta_{2}$ for all 81 simulation scenarios. Regression coefficient $\beta_{2}$ was estimated by maximizing the likelihood (ML), by maximizing Firth’s penalized likelihood (FL), by exact Poisson regression (EP) and by using Bayesian data augmentation (BDA). Rows correspond to the number of covariates in the respective simulation scenario, columns to the events per variable ratio and ticks on the x-axis to the true value of $\beta_{1}$. Grey step functions below the plots indicate sample size.


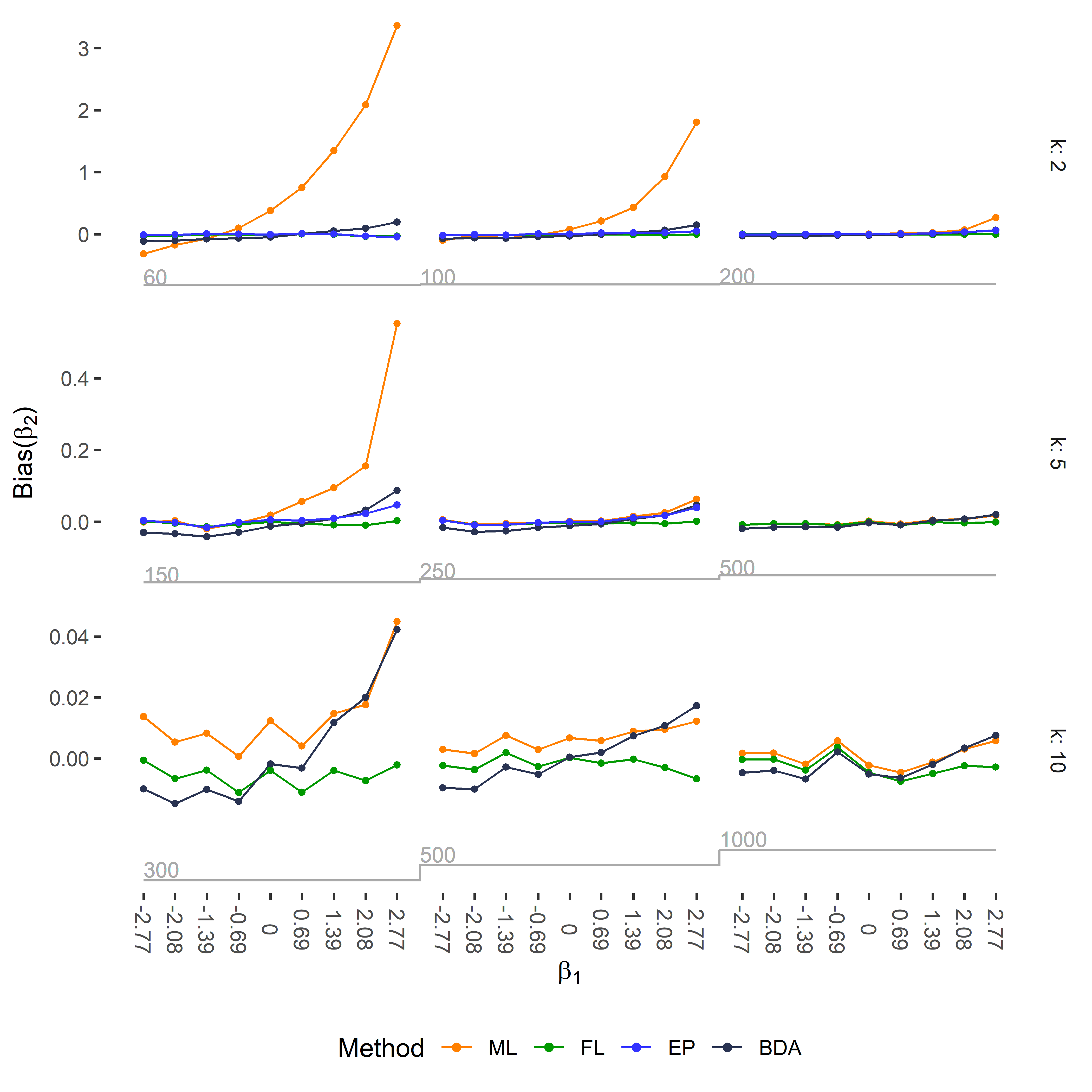


Figure S6: Relative frequency of 95% two-sided confidence intervals excluding $\beta_{1}=0$ for all 81 simulation scenarios. For simulation scenarios with $\beta_{1}\neq0$, this describes the power. For scenarios with $\beta_{1}=0$, the relative frequency of intervals excluding $0$ is equal to 5% (marked by the dotted line), given the interval is maintaining its coverage probability at the nominal level of 95%. Confidence intervals were estimated using the Wald method with maximum likelihood estimation (ML), likelihood profiles with Firth’s penalized likelihood estimation (FL), exact interval estimates (EP) and mid-p corrected confidence intervals (EP:mid-p) with exact Poisson regression and likelihood profiles with Bayesian data augmentation (BDA). Rows correspond to the number of covariates in the respective simulation scenario, columns to the events per variable ratio and ticks on the x-axis to the true value of $\beta_{1}$. Grey step functions below the plots indicate sample size.


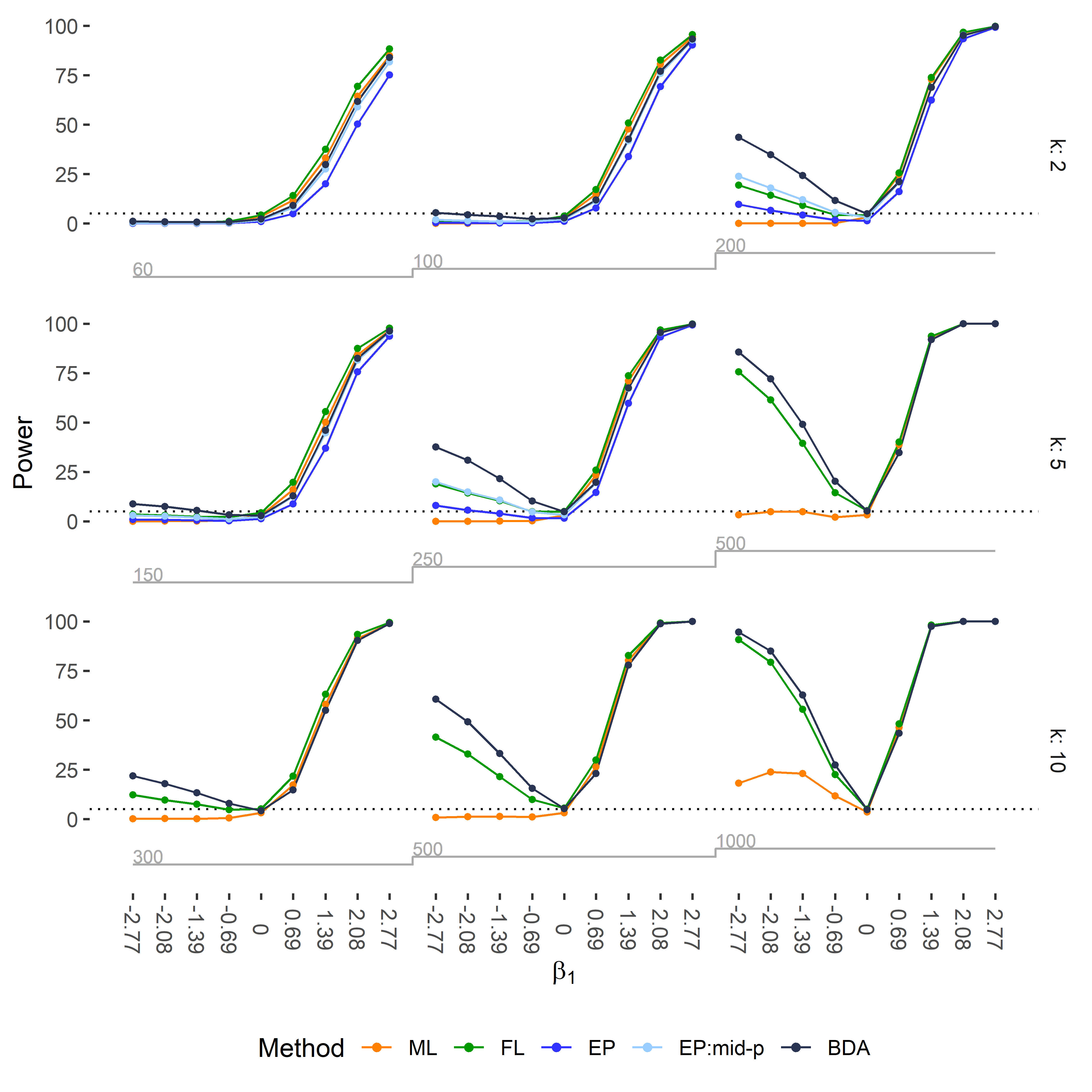


Figure S7: Median width of 95% two-sided confidence intervals for all 81 simulation scenarios. Confidence intervals were estimated using the Wald method with maximum likelihood estimation (ML), likelihood profiles with Firth’s penalized likelihood estimation (FL), exact interval estimates (EP) and mid-p corrected confidence intervals (EP:mid-p) with exact Poisson regression and likelihood profiles with Bayesian data augmentation (BDA). Rows correspond to the number of covariates in the respective simulation scenario, columns to the events per variable ratio and ticks on the x-axis to the true value of $\beta_{1}$. Grey step functions below the plots indicate sample size. For some scenarios the median width of ML-Wald intervals, exact intervals and mid-p corrected intervals exceeded the plotting range of the Figure.


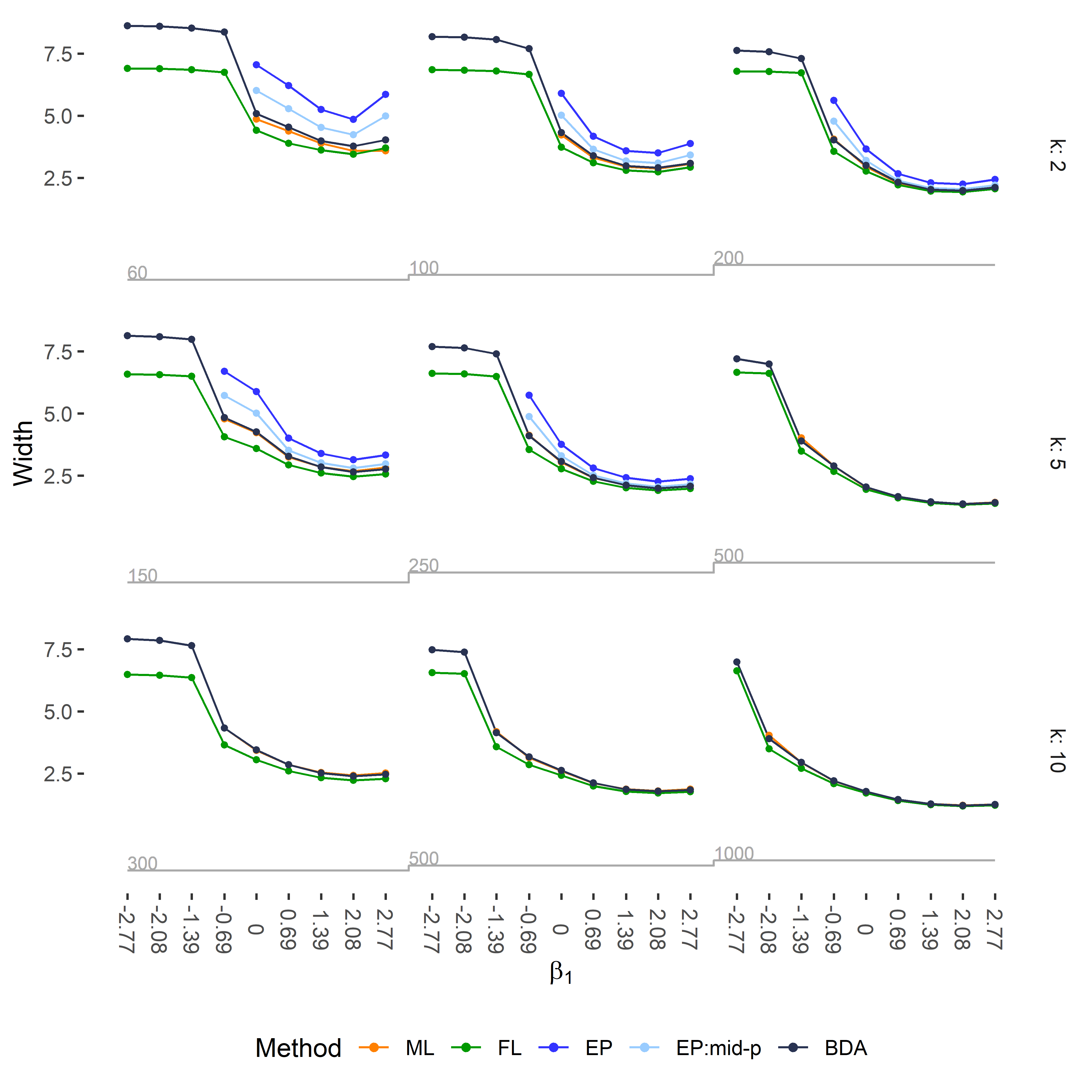


Table S1. Implant dentistry study: baseline characteristics, number of implantations and number of hematologic complications.

| Variable | Count (per cent) |
| --- | --- |
| Age:*  20  30  40  50  60  70  80  90 | 88 (7.8)  128 (11.3)  176 (15.6)  260 (23.0)  238 (21.0)  157 (13.9)  74 (6.5)  11 (1.0) |
| Smoking:  No  Light  Heavy | 912 (80.8)  157 (13.9)  60 (5.3) |
| Diabetes Mellitus:  absent  present | 1099 (97)  34 (3) |
| Number of implantations:  1  2  3  4  >4 | 524 (46.3)  311 (27.5)  95 (8.4)  125 (11.0)  78 (6.8) |
| Hematological complications:  0  1  2  4 | 1,121 (98.9)  3 (0.3)  1 (0.1)  8 (0.7) |

* for data privacy reasons, age was rounded to the nearest multiple of 10
